# Novel, non-invasive technique for fracture reduction in rare pertrochanteric femur fractures in patients with ipsilateral above-knee amputation: a technical report

**DOI:** 10.1007/s00068-025-03078-0

**Published:** 2026-02-02

**Authors:** Aditya Vadgaonkar, Michael Hackl, Sascha Gravius, Ali Darwich, Alexander Blümke, David Eschmann, Frederic Bludau

**Affiliations:** 1 Department of Orthopaedic and Trauma Surgery, University Medical Centre Mannheim, Medical Faculty Mannheim, University of Heidelberg, Theodor-Kutzer-Ufer 1-3, 68167 Mannheim, Germany; 2 Department of Orthopaedic and Trauma Surgery, Marienhaus Hospital Hetzelstift, Stiftstraße 10, 67434 Neustadt an der Weinstraße, Neustadt, Germany

## Abstract

**Introduction:**

Pertrochanteric femur fractures in patients with ipsilateral above-knee amputations present unique challenges, as the use of standard reduction techniques such as traction tables is impractical. Modified techniques, including the use of K-wires, Schanz pins, or bone clamps, are often invasive and carry the risk of vascular disruption and surgical site infections. Given these limitations, we developed a non-invasive technique that leverages the cephalomedullary nail as a joystick to reduce the fracture.

**Methods:**

Patients with a history of ipsilateral above-knee amputation who subsequently sustained a pertrochanteric femur fracture were treated using the novel technique at our institution between January 2019 and January 2024. Radiological outcomes in these patients were evaluated by assessing fracture reduction quality according to Schipper et al. and fixation quality using the Cleveland and Bosworth quadrant system. The results are summarised in this technical report.

**Results:**

Between January 2019 and January 2024, three patients (two males, mean age 84 ± 11 years) with an ipsilateral above-knee amputation were treated using the novel technique with a cephalomedullary nail using TFN-Advanced™ Proximal Femoral Nailing System or PFNa, Proximal Femur Nail Antirotation (DePuySynthes, Johnson & Johnson MedTech). These patients had a mean Charlson Comorbidity Index of 10± 2.1. Radiological evaluation showed anatomic reduction in two cases and acceptable reduction in one, with optimal femoral neck fixation in two cases. The mean operative time and hospital stay were 89 ± 100 min and 18 ± 15 days respectively. No surgery-related complications occurred, and all patients were mobilised with full weight-bearing using their exoprostheses.

**Conclusion:**

This novel technique provides a safe alternative for fracture reduction in amputees, avoiding the risks associated with invasive hardware. It offers advantages such as minimal soft tissue disruption, which might subsequently improve patient outcomes. Further studies with larger cohorts are necessary to validate its efficacy and determine its broader applicability in clinical practice.

## Introduction

Global trends suggest an increase in the number of lower limb amputees, with diabetes being the most common cause of lower-limb amputation [1]. Following amputation, altered biomechanics, including changes in gait and movement patterns, combined with a lack of sensory feedback, significantly impair balance and increase the risk of falls [2]. Survey data indicate that more than half of lower-limb amputees experience at least one fall annually [3]. Patients with lower-limb amputation are also prone to loss of bone mineral density due to abnormal or reduced loading on the femur [4, 5]. Low bone mass and microarchitectural deterioration of bone due to osteoporosis are the greatest risk factors for hip fractures, as soft and fragile bone in patients with osteoporosis is susceptible to fracturing even with low-energy trauma [6]. Current evidence suggests that the incidence of proximal femur fractures is high in amputees, with a trajectory that mirrors the rising prevalence of hip fractures in the general population [7–9]. 

The standard approach for treating per- and intertrochanteric types of proximal femur fractures involves closed reduction and internal fixation (CRIF) using cephalomedullary nailing systems, as recommended by various national orthopaedic and trauma societies [10, 11]. By providing stable fixation, this allows for early mobilization and weight-bearing, both of which are critical for minimizing complications and promoting optimal postoperative recovery [12]. These systems typically rely on the use of a traction table, which enables the surgeon to apply controlled longitudinal traction to achieve preliminary fracture reduction [13]. However, the presence of an intact distal limb segment is crucial for patient positioning, making the use of a traction table impractical in patients with ipsilateral above-knee amputations.

To overcome this challenge, various alternatives have been proposed, including the use of semi-invasive methods such as the insertion of Kirschner wires, Schanz pins or bone clamps [14–17]. These methods involve additional drilling into the distal portion of the amputation stump to facilitate manipulation of the fracture site. However, amputation stumps are inherently predisposed to complications such as inflammation, infection, and, in rare cases, malignancy [18]. The additional drilling required for these techniques can cause microvascular damage and disrupt local blood flow, further increasing the risk of infection and other complications [19]. Other non-invasive methods, such as the use of fabric tape, have also been described. However, these approaches are often unstable and may not provide the precision required for adequate fracture reduction [20, 21]. 

Given these limitations, we sought to develop a novel, non-invasive, and stable technique for fracture reduction that leverages the cephalomedullary femoral nail itself, eliminating the need for additional hardware and minimizing the risk of complications and is described in the present technical report.

## Methods

Patients presenting to our hospital with a pertrochanteric fracture and an ipsilateral AK amputation were managed using the new technique. All procedures were performed using a cephalomedullary implant (TFN-Advanced™ Proximal Femoral Nailing System, DePuy Synthes, Johnson & Johnson MedTech or PFNa, Proximal Femur Nail Antirotation, DePuy Synthes, Johnson & Johnson MedTech).

### Inclusion criteria

All patients presenting to our tertiary-level trauma centre between January 2019 and January 2024 with a pertrochanteric femur fracture (AO Types 31A1, A2, and A3) [22] and a history of ipsilateral transfemoral amputation were considered eligible. Inclusion required that the amputation had been performed at least two years prior to injury and that the stump was fully healed at the time of presentation. All patients meeting these criteria were managed using the novel, non-invasive reduction technique. No exclusion criteria were applied based on age or comorbidity burden.

### Surgical technique (Step-by-Step Description)

All procedures were performed by experienced trauma surgeons with the patient positioned supine on a radiolucent operating table. The unaffected limb was placed in a standard lithotomy position to allow unobstructed fluoroscopic imaging. A traction table was not used. All procedures were performed using a cephalomedullary implant. Either TFN Advanced™ (Trochanteric Femoral Nailing System Advanced, DePuy Synthes, Johnson & Johnson MedTech) or PFNa (Proximal Femur Nail Antirotation, DePuy Synthes, Johnson & Johnson MedTech) systems were used.

#### Patient Preparation and positioning

The ipsilateral above-knee amputation stump was carefully padded, disinfected, and draped circumferentially to allow unrestricted access throughout the procedure.

#### Application of traction via the stump

**Fig. 1:**
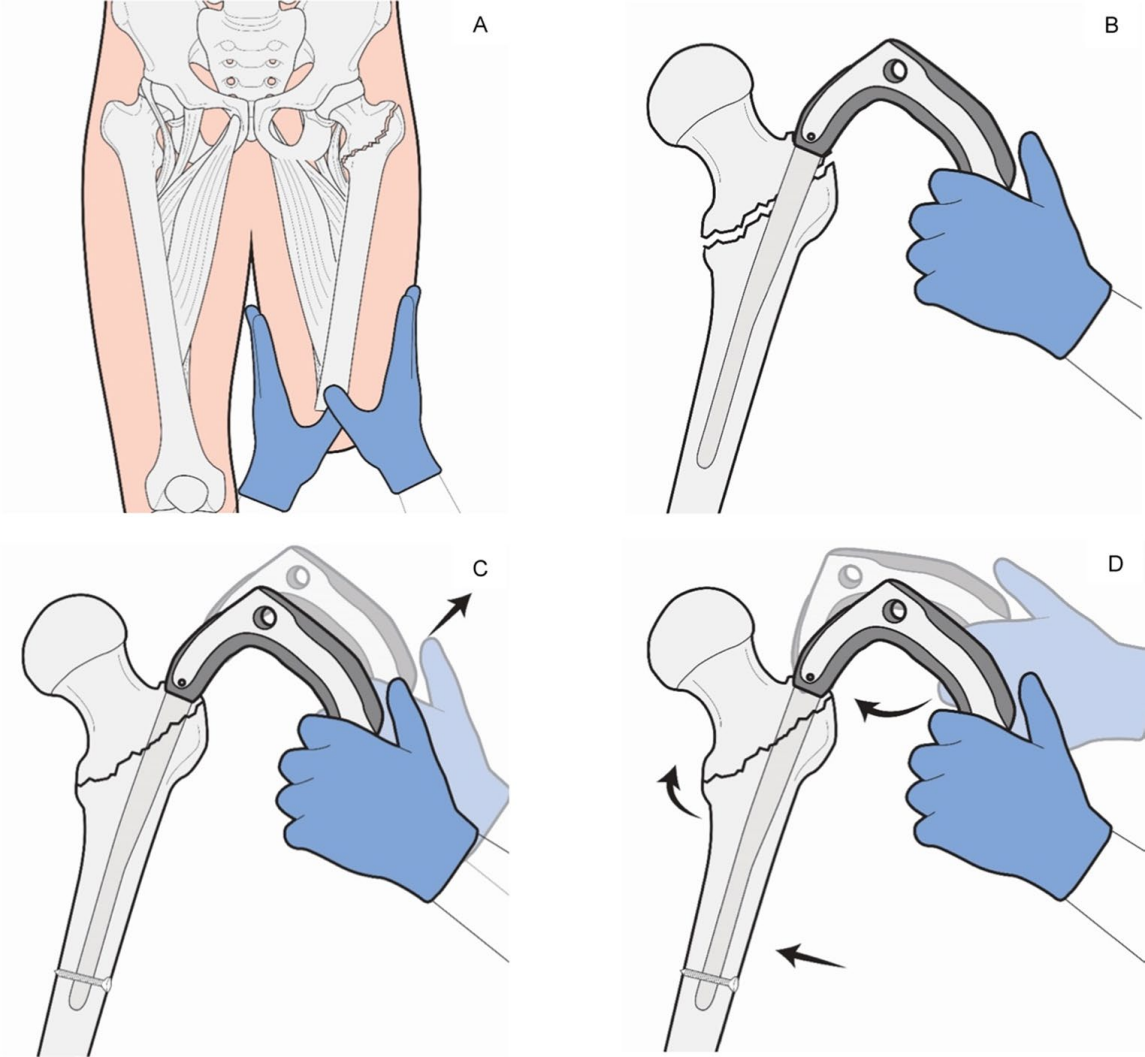
Reposition technique using the CMN as a joystick. **A**: After prepping and draping the stump to allow full access, longitudinal manual traction is applied along the axis of the femur to restore length and obtain gross rotational alignment while an assistant maintains steady traction throughout. **B**: The CMN is inserted at the tip of the greater trochanter as usual and advanced to the desired working depth (**C**): Distal locking is performed first, converting the nail–distal fragment construct into a controlled fulcrum. With the distal screw acting as a pivot, the distal fragment can be manipulated relative to the proximal fragment by gently elevating or depressing the nail handle to achieve cortical alignment. (**D**): Fine adjustments in alignment are made by tilting the nail handle medially or laterally to correct varus or valgus deformity in the coronal plane and by rotating the handle anteriorly or posteriorly to correct anteversion or retroversion in the transverse plane. Once satisfactory reduction is confirmed fluoroscopically in both planes, proximal locking and insertion of the helical blade are performed as usual

Longitudinal manual traction was applied along the anatomical axis of the femur by an assistant. Traction was achieved by circumferentially grasping the stump using both hands over padded sterile towels, ensuring uniform force distribution and avoiding localised pressure on the skin or distal soft tissues (Fig. 1A). This manoeuvre was used to restore femoral length and achieve gross rotational alignment. Traction was maintained steadily during the initial steps of nail insertion.

#### Nail insertion

The entry point of the nail was at the tip of the greater trochanter under fluoroscopic control. A guidewire was inserted and confirmed in both AP and lateral planes. The medullary canal was prepared and a cephalomedullary nail was advanced across the fracture site to a controlled working depth under fluoroscopy (Fig. 1B).

#### Distal locking

Contrary to the conventional sequence, distal locking was performed first using static locking. This step rigidly coupled the nail to the distal fracture fragment, effectively converting the nail–distal fragment construct into a stable lever arm. At this stage, the nail was not yet fixed proximally, allowing controlled relative movement between the distal and proximal fragments (Fig. 1C).

#### Fracture reduction using the nail as a joystick

With distal locking completed, fracture reduction was achieved through deliberate manipulation of the nail handle under fluoroscopic control. In the coronal plane the varus-valgus alignment was corrected by tilting the nail handle medially or laterally, adjusting the neck–shaft angle. In the transverse plane anteversion-retroversion Rotational alignment was refined by rotating the nail handle anteriorly or posteriorly. Minor adjustments in length and translational alignment were achieved through gentle axial traction and controlled elevation or depression of the nail handle (Fig. 1D). This allowed precise, multiplanar fracture reduction.

#### Proximal locking and final fixation

Once satisfactory reduction was confirmed on both AP and lateral fluoroscopic views, proximal fixation was completed by insertion of the helical blade or lag screw according to standard technique. Final fluoroscopic imaging confirmed implant position, fracture alignment, and appropriate blade placement, before final wound closure as per standard protocol.

### Evaluation of patient characteristics and radiological outcomes

The preoperative comorbidity burden of the patients was calculated using the Charlson Comorbidity Index (CCI) [23]. The radiological outcomes were evaluated on the basis of the quality of fracture reduction and fixation via anteroposterior (AP) and lateral radiographs obtained on the second postoperative day. Reduction quality was assessed on the basis of the angular orientation of the femoral head in the transverse and coronal planes, using the criteria described by Schipper et al.^24^ Reduction was categorized as anatomic if there was perfect cortical alignment without any deviation in the neck-shaft angle, acceptable if there was a deviation of 5°–10° in varus/valgus and/or anteversion/retroversion, and poor if the deviation exceeded 10°. Fixation quality was evaluated by determining the position of the neck screw within the femoral neck and head according to the Cleveland and Bosworth quadrant system [25]. 

## Results

### Patient demographics and fracture characteristics

Between January 2019 and January 2024, three patients (two males, mean age 84 ± 11 years) presented with a pertrochanteric femur fracture (AO Type 31A1, 31A2, 31A3) [22] and had a history of ipsilateral transfemoral amputation with a fully healed stump. All these patients were successfully treated with our novel technique. This group had a Charlson comorbidity index of 10 ± 2.1.

### Duration of surgery

The mean operative time, defined as the time from the first skin incision after patient positioning to wound closure, was 89 ± 100 min. All the patients could be successfully treated with the new technique without the need for conversion to open reduction.

### Radiological outcomes

**Fig. 2:**
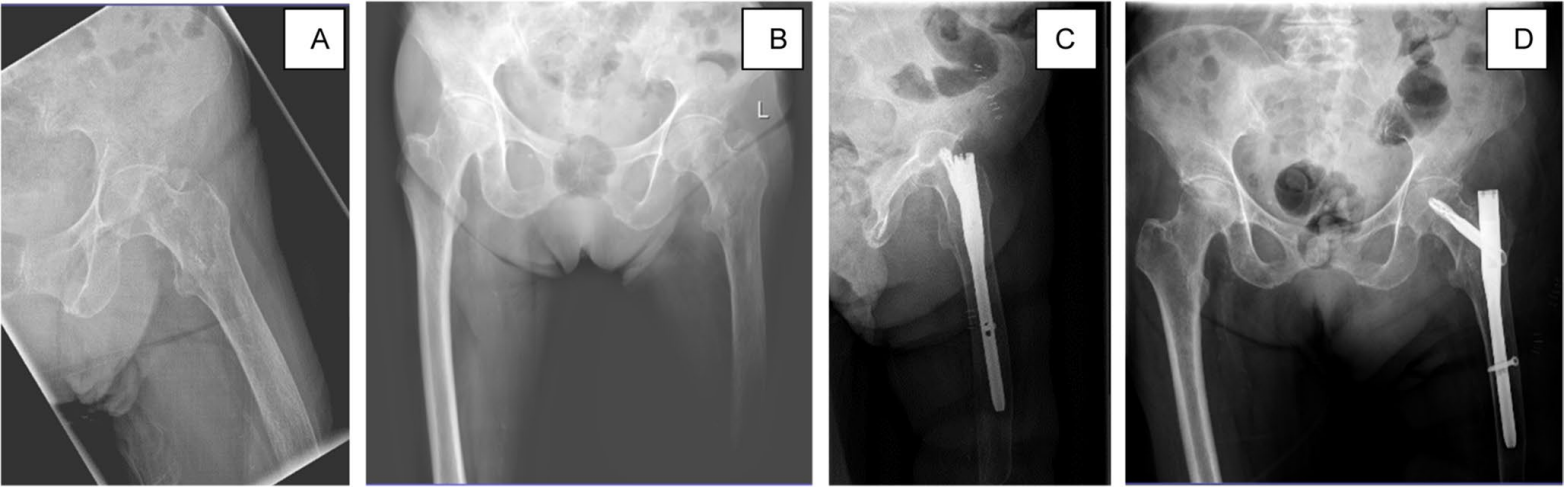
Preoperative radiographs showing a left pertrochanteric femur fracture (**A**, **B**). Postoperative radiographs showing position of the cephalomedullary nail (**C** and **D**)

Radiological evaluation of reduction quality using postoperative radiographs revealed that anatomic reduction was achieved in two cases and acceptable reduction was achieved in one case, with optimal femoral neck fixation in two patients per the definitions mentioned earlier (Fig. 2).

### Postoperative complications and hospital stay

No cases of superficial or deep surgical site infection were observed, and none of the patients required revision surgery or implant removal. All patients were mobilised with full weight-bearing on the affected leg with the help of their exoprosthesis beginning on the first postoperative day. One patient died during the hospital stay due to a paraneoplastic embolism, which was unrelated to the surgical procedure. The mean hospital stay was 18 ± 15 days for amputees vs. 13.6 ± 8.8 days for non-amputees.

## Discussion

Lower limb amputation represents a significant health burden, profoundly affecting mobility and quality of life [26]. Amputees face numerous psychosocial and health challenges, including an increased risk of falls, which predisposes them to fractures [7]. Proximal femur fractures, in particular, present a unique surgical challenge in amputees owing to the difficulty of stump manipulation during the procedure. Although no standardised guidelines exist for the management of proximal femur fractures in above-knee amputees, evidence suggests that with appropriate surgical techniques, good outcomes can be achieved, allowing patients to regain mobility comparable to their preinjury status [27]. Additionally, research indicates that fractures in amputated limbs behave similarly to those in intact extremities, with osteoporosis showing no negative effect on fracture healing or callus formation, highlighting the critical role of surgical management [28]. The primary challenge in the surgical management of hip fractures in above-knee amputees lies in the absence of a distal limb segment for anchoring, which renders the standard traction tables useless. To address this, a few alternative methods have been proposed, but each has significant limitations that restrict their effectiveness and applicability.

One widely used approach involves inserting a Kirschner wire distally into the amputation stump under fluoroscopic guidance and attaching a horseshoe holder or an Illizarov half ring to create a fixation point for traction and manipulation of the fracture fragments [16, 17, 29]. While this method provides good traction, the authors report a lack of rotational control. Jain et al. proposed the use of Schanz pins instead of K-wires to improve stability; however, their study reported inadequate reduction, necessitating open reduction via an anterior approach [30]. Berg and Bhatia described the use of a Steinmann pin inserted distally, connected to a traction bow and a series of clamps to enable rotational control [31]. In this case, minimal traction was required due to negligible limb shortening, and the fracture was identified only on MRI. While this method offers rotational control, it cannot sustain substantial traction due to the risk of pin cut-out, particularly in osteoporotic bone, which is common in amputees.

To mitigate the risk of cut-out using the Steinmann pin, Patwardhan et al. suggested the use of a Denham pin with central threads to achieve better bone purchase and improve hold. This modification allowed for adequate axial traction, with rotational adjustments performed by the assistant by rotating the stirrup through sterile drapes [32]. Nardulli and Issack suggested the use of two sets of two Schanz pins and attaching them to an external AO femoral distractor, thus eliminating the need for a traction Table [15] While effective, this technique requires careful rotational alignment prior to pin insertion, along with the use of an obturator outlet, an obturator inlet, and oblique iliac views on fluoroscopy to ensure accurate pin placement in the anterior inferior iliac spine, making the procedure slightly technically demanding and time-consuming.

Despite their effectiveness, all these methods are more invasive, requiring the drilling of additional pins into the stump. This increases the risk of neurovascular injury and disrupts the vascular integrity of the stump, potentially leading to microvascular damage and compromised blood flow. ^33^ These complications are particularly concerning in diabetic amputees, who are predisposed to delayed wound healing and surgical site infections, often hindering the use of an exoprosthesis. ^34^

Non-invasive methods of fracture reduction advocate the use of fabric or skin tape to apply axial traction. ^20,21^ In this technique, tape is wrapped circumferentially around the distal end of the amputation stump, and axial traction is applied. While this method avoids the invasiveness of pin insertion, it is inherently unstable and permits only limited axial traction. Rotational control is minimal, restricting its application to fractures with minimal rotational displacement. Additionally, the delicate skin around the stump is particularly susceptible to injury during prolonged traction, increasing the risk of skin breakdown.

Our non-invasive method leverages the inserted nail itself as a reduction tool, thus eliminating the need for a traction table. Recent studies, including a meta-analysis, have shown no significant advantage of traction tables in improving patient outcomes, highlighting that good fracture reduction can be achieved while saving operative time when manual traction is used [35, 36]. In our method, the cephalomedullary nail was locked distally, over which the distal fragment could be manipulated relative to the proximal fragment, allowing fine adjustments in length, alignment, and rotation. The technique is straightforward to implement and does not require specialised equipment. A conceptually related technique has been described previously, in which controlled “backstroking” of an intramedullary implant after distal locking is used to generate intrafragmentary compression such as in the case of a tibial shaft fracture.^37^ This approach of using the implant itself to reduce the fracture eliminates the need for invasive hardware and preserves the vascular integrity of the amputation stump. Additionally, it eliminates the additional steps of pin insertion and removal. However, the mean operative time for the novel technique was longer than that of the standard procedure, which likely reflects the inherent complexity of operating on amputee patients and the initial learning curve associated with introducing a new method. The increased time may also be attributed to patient positioning challenges and the need for careful intraoperative adjustments in the absence of a traction table.

While this technique offers several advantages, certain practical considerations must be acknowledged. Rotational alignment should be roughly established before nail insertion, as adjustment is limited once distal locking has been performed. The method also relies on manual traction by an assistant to maintain limb length and alignment during reduction. In addition to these technical factors, the main limitation of this study is its small sample size. The study corresponds to stage 2a of the IDEAL framework and is intended to demonstrate the feasibility and safety of the novel technique in a rare and complex clinical setting.^38^ Larger, prospective, multicentre studies are warranted to validate these preliminary results and further define the technique’s clinical applicability.

## Conclusion

This novel, non-invasive technique offers a practical and less invasive alternative for achieving fracture reduction in patients with an ipsilateral above-knee amputation. By eliminating the need for additional hardware, it minimizes the risk of vascular and soft tissue complications associated with traditional pin-based methods. The present findings highlight the feasibility and safety of this approach in a small cohort of patients. While these preliminary results are encouraging, larger, prospective studies are needed to further validate the technique and better define its role within the broader context of pertrochanteric fracture management.

## Data Availability

The dataset analysed during the current study are not publicly available due to institutional privacy policies and patient confidentiality regulations. However, anonymized data may be made available from the corresponding author upon reasonable request and with permission from the institutional ethics committee. Anonymised pre- and postoperative radiograph from one of the patients has been included in the manuscript for illustrative purposes (Figure 2).
